# Drug titration patterns and HbA_1c_ levels in type 2 diabetes

**DOI:** 10.1111/j.1742-1241.2009.02094.x

**Published:** 2009-07

**Authors:** J Ross Maclean, R H Chapman, C P Ferrufino, G Krishnarajah

**Affiliations:** 1 Bristol-Myers SquibbPrinceton, NJ, USA; 2 US Health Economics and Outcomes Research, IMS Health, Falls ChurchVA, USA

## Abstract

**Objective:**

To evaluate oral antidiabetes drug (OAD) use, haemoglobin A_1c_ (HbA_1c_) testing and glycaemic control in type 2 diabetes patients.

**Study design:**

Retrospective analysis based on claims data from the Integrated Healthcare Information Services (IHCIS) National Managed Care Benchmark Database.

**Methods:**

OAD use and HbA_1c_ testing were analysed for patients with ≥ 2 claims indicating diagnosis of type 2 diabetes and ≥ 1 90-day OAD treatment period between 1 January, 2000 and 30 June, 2006. Likelihood of HbA_1c_ testing was examined using multivariable logistic regression analyses, adjusting for OAD regimen and patients’ sociodemographical characteristics.

**Results:**

Patients were classified based on initial OAD regimen: metformin (MET) (*n* = 22,203; 41.3%), sulphonylurea (SFU) (*n* = 18,439; 34.3%), thiazolidinedione (TZD) (*n* = 7663; 14.3%), SFU + MET (*n* = 5467; 10.2%) and TZD + MET (*n* = 2355; 4.2%). A total of 51.5% of patients had HbA_1c_ testing during 90 days preceding OAD initiation through regimen completion. Approximately, 65% of MET and 58% of SFU patients had no titration of initial regimen. Patients demonstrating inadequate glucose control decreased from 68.5% at baseline to 46.9% within 90 days of regimen initiation. Multivariable logistic regression indicated several negative predictors of HbA_1c_ testing, including SFU use, age 65+ years, moderate insurance copayment and preindex inpatient utilisation. Multivariable logistic regression of variables associated with reduced likelihood of up-titration included TZD, SFU + MET, or TZD + MET treatment, age 18–34 years, Medicare insurance and any preindex healthcare utilisation.

**Conclusions:**

Patients are not being transitioned to additional OADs in a stepwise fashion and/or are receiving inadequate titration on current OAD regimens. The low rate of HbA_1c_ testing and rates of control are contributing factors.

**What’s known**

- The availability of multiple pharmacological agents has extended the duration of time during which patients with type 2 diabetes can maintain glycaemic control using oral antidiabetes drugs (OADs) alone.
- Research has shown, however, that even in well-managed healthcare organisations that follow standardised treatment protocols, patients with inadequate glycaemic control frequently experience suboptimal management of OAD treatment regimens, in particular, and delays in therapeutic transitions or up-titrations. Furthermore, evidence has indicated that HbA_1c_ testing is substantially underutilised, despite the current American Diabetes Association (ADA) recommendation for biannual HbA_1c_ measurements, with more frequent testing (every 3 months) when glucose levels are not well controlled.

**What’s new**

This large claims analysis examined OAD regimen usage patterns, as well as haemoglobin A_1c_ (HbA_1c_) testing frequency and control, among a large population of patients with type 2 diabetes.

- Only 52% of patients received an HbA_1c_ test at any time during their OAD regimen. Many (68.5%) demonstrated inadequate glucose control in the 90 days following OAD initiation. Only a minority (32.5%) received any OAD titrations during treatment.
- The results of this study verify that inadequate HbA_1c_ testing and control, as well as a lack of timely, stepwise OAD transitions and/or titrations are common in the US.

## Introduction

Diabetes is a disease of elevated blood glucose related to inadequate insulin production and/or utilisation. The progressive β-cell failure associated with type 2 diabetes contributes to the inevitable deterioration of glucose control over time and the need for increasingly aggressive treatment regimens (1,2). Diabetes represents a growing worldwide epidemic and is a major global health and economic concern. Evidence has indicated that both the prevalence and incidence of diabetes are on the rise, with both increasing by approximately 5% annually in the US over the past 15 years (3,4).

The availability of multiple pharmacological agents has extended the duration of time during which patients with type 2 diabetes can maintain glycaemic control using OADs alone (5). The likelihood of success with any OAD management strategy, however, is dependent on factors such as patient lifestyle modifications and adherence, physician adherence to guidelines and stepped prescribing patterns (5–10). With recent evidence indicating the potential benefit of more aggressive, stepwise therapy in type 2 diabetes, a number of algorithms have been published to facilitate timely treatment transitions in response to persistently elevated glucose levels (5,11,12). Research has shown, however, that even in well-managed healthcare organisations that follow standardised treatment protocols, patients with inadequate glycaemic control frequently experience suboptimal management of OAD treatment regimens, in particular, delays in therapeutic transitions or up-titrations (7,13,14).

HbA_1c_ is currently the standard serum marker applied to assess overall glycaemic control in patients with diabetes. Although national guidelines agree that targeting an HbA_1c_ level of < 7% or even lower is desirable for the majority of patients (15,16), HbA_1c_ control remains elusive for most patients. Results from one national survey conducted in 2004 revealed that 73% of individuals with type 2 diabetes had HbA_1c_ levels that exceeded target (17). Furthermore, evidence indicated that HbA_1c_ testing is substantially underutilised, despite the current ADA recommendation for biannual HbA_1c_ measurements with more frequent testing (every 3 months) when glucose levels are not well controlled (16). The most recent data available from the US Centers for Disease Control’s Behavioral Risk Factor Surveillance System (BRFSS) survey indicated that in 2005, only 64.3% of patients with diabetes self-reported at least 2 HbA_1c_ tests in the preceding year (18). A registry audit and a study of a large managed care population confirmed these findings, indicating that when lacking any intervention to encourage testing, only approximately 50% of patients received at least one HbA_1c_ test over 6-month and 1-year periods (19,20).

Thus, existing evidence points to inadequacies in both the OAD management and HbA_1c_ monitoring strategies that are currently applied in clinical practice. It is likely that both of these factors contribute to suboptimal glycaemic control in patients with type 2 diabetes. The following claims analysis study was designed to validate these hypotheses through an examination of usage patterns for specific OAD regimens, as well as HbA_1c_ test utilisation and outcomes, among a large population of patients with type 2 diabetes.

## Research design and methods

### Study population

The population for this retrospective analysis was derived from health insurance claims data and enrolment records for approximately 40 US health plans using information obtained from the Integrated Healthcare Information Services (IHCIS) National Managed Care Benchmark Database. Initial database screening led to the identification of 916,211 individuals with ≥ 2 claims for diabetes mellitus (ICD-9-CM code 250.xx) during the study period (1 January, 2000, through 30 June, 2006). Members of this group were eligible for study inclusion if they had: (i) a diagnosis of type 2 diabetes (ICD-9-CM code 250.x0 or 250.x2 in any listed diagnosis field) on ≥ 2 claims during the study period; (ii) had undergone at least one continuous 90-day period of prescribed OAD therapy; (iii) were aged ≥ 18 years at the earliest OAD fill date; and (iv) exhibited an absence of documented OAD pharmacy claims in the 180-day period preceding first OAD treatment during the study period. Individuals with no OAD use during the study period, non-continuous enrolment in an included health plan during the OAD treatment period, or a diagnosis of type 1 diabetes (ICD-9-CM code 250.x1 or 250.x3) or gestational diabetes (ICD-9-CM code 648.8) on ≥ 1 claim(s) during the study period were excluded from analysis.

### Oral antidiabetes drug treatment regimens

OAD utilisation histories for the study population were derived from IHCIS pharmacy claims data. An OAD regimen was defined as a single prescription for OAD monotherapy or single-pill combination therapy, or prescriptions for ≥ 2 OADs combined as dual therapy, in which sufficient medication was provided for a treatment period of ≥ 90 days. Dual-therapy prescriptions were required to be filled within 25 days of one another, to ensure that there was overlapping supply of the two medications. The start date for a dual-therapy regimen was considered to be the date on which the second OAD prescription was initially filled. New OAD prescriptions were distinguished from refills by a ≥ 180-day clean period, during which time a patient had no prescriptions filled for any OAD, but remained continuously enrolled in an included health plan.

For each patient, the OAD regimen with the earliest start date was referred to as their index regimen. The preindex period was defined as the 180-day period preceding the start date of an index regimen. The observation period extended from the start of the preindex period through disenrolment or study termination. An OAD regimen was considered ongoing as long as there was no gap in treatment coverage (based on daily medication requirements) of > 120 days for any OADs in the regimen, and provided that at least one prescription refill was available for all OADs in the regimen. The end of a regimen occurred when ≥ 1 OAD(s) were added to and/or subtracted from the regimen. Subtraction of an OAD was defined as a gap in treatment coverage of > 120 days for ≥ 1 OAD(s) in an otherwise ongoing regimen. Resumption of a treatment regimen following a gap of > 120 days was considered equivalent to the initiation of a new regimen. Censoring of a treatment regimen referred to regimen truncation because of patient disenrolment from an included health plan or study termination.

OADs were classified into six groups: sulphonylureas (SFU), non-sulphonylurea insulin secretagogues (NIS), metformin (MET), thiazolidinediones (TZD), alpha-glucosidase inhibitors and other. Appendix S1 provides a list of medications associated with each of these OAD classes.

### OAD titrations and treatment gaps

Data on OAD up-titrations and down-titrations were captured for analysis. Information included: (i) the total numbers of each type of titration during each OAD regimen; (ii) determination of whether HbA_1c_ testing was performed during the 90-day period preceding and/or the 90-day period following each titration; and (iii) characterisation of patients’ pretitration and posttitration glucose control levels based on HbA_1c_ test results. Gaps in OAD treatment of > 3 days and ≥ 30 days were also noted. Cross-tabulation analysis was performed to determine the association between OAD regimen type and titration patterns and between OAD regimen type and treatment gaps.

### HbA_1c_ testing

HbA_1c_ testing patterns and results were captured for analysis. Information included: (i) the number of patients undergoing HbA_1c_ testing; (ii) the number of HbA_1c_ tests performed during each OAD regimen; (iii) assessment (yes/no) of whether HbA_1c_ testing was performed during the 90-day period preceding and/or the 90-day period following the start date of each regimen and each OAD up-titration and down-titration; (iv) assessment (yes/no) of whether HbA_1c_ testing was performed during the 90-day \period prior to the end date of each regimen; and (v) an evaluation of HbA_1c_ levels obtained prior to and throughout the course of each OAD regimen. Cross-tabulation analysis was performed to determine the association between OAD regimen type and HbA_1c_ test results.

### Glucose control

Glucose control was assessed based on the results of HbA_1c_ testing. HbA_1c_ results were categorised based on currently accepted ADA and Healthcare Effectiveness Data and Information Set (HEDIS) classifications of blood glucose levels as normal (< 6%, used for sensitivity analyses only), controlled (< 7.0%), suboptimally (7.0% to 9.5%) or poorly controlled (≥ 9.5%) (16,21). Note that, at the time of this analysis, the IHCIS database contained data on laboratory values for *only approximately 2%* of all patients. (In contrast, laboratory *claims* data were available for all patients.) The level of glucose control was examined on the subset of patients for whom laboratory value data were available [MET, *n* = 2946 (13.3%); SFU, *n* = 2785 (15.1%); TZD, *n* = 1253 (16.4); SFU + MET, *n* = 754, (13.8%); TZD + MET, *n* = 321 (13.6%)].

### Statistical analysis

In addition to the cross-tabulation analyses previously described, multivariable logistic regression analyses were performed to model which characteristics were predictive of patients receiving any HbA_1c_ testing (binary variable: yes/no). Separate logistic regression analyses modelled the factors associated with greater likelihood of up-titration of the index OAD regimen. Variables included were OAD treatment, patient age, gender, US region, insurance type, amount of copayment, insulin use (yes/no), inpatient, outpatient, laboratory or other services and time to first OAD after diabetes diagnosis. Analyses were performed using SAS Institute Inc. software (SAS ver. 9.1.3, Cary, NC, USA).

## Results

### Index OAD regimen groups

A total of 420,329 patients satisfied the screening criteria. Although treatment data were collected for all prescribed OADs, only the most frequently occurring index OAD regimens in the screened population were selected for analysis. After excluding combination regimens consisting of medications from three or more OAD classes, the distribution of patients for each of the selected index regimens was: MET, *n* = 22,203 (39.6%); SFU, *n* = 18,441 (32.9%); TZD, *n* = 7663 (13.7%); SFU + MET, *n* = 5467 (9.7%) and TZD + MET, *n* = 2356 (4.2%).

### Sociodemographical and healthcare profiles

Analysis of the resulting patient population included an examination of sociodemographical characteristics, healthcare utilisation, health insurance and treatment histories by index OAD regimen. There were few differences between index regimen groups with regard to gender, age, geographical region, health insurance type and healthcare expenditures during the preindex year (Table 1).

**Table 1:** Patient sociodemographical characteristics and healthcare profiles

	Index OAD regimen					
Parameter	MET(*n* = 22,203)	SFU(*n* = 18,441)	TZD(*n* = 7663)	SFU + MET(*n* = 5467)	TZD + MET(*n* = 2356)	Total(*n* = 56,130)
**Gender**						
Female (%)	46.9	44.6	44.9	45.6	44.3	45.6
Average age (years) (SD)	56.8 (12.1)	57.3 (12.2)	56.6 (11.9)	56.0 (12.0)	57.0 (12.3)	56.9 (12.1)
**Age group (years) (%)**						
18–34	3.8	3.5	3.6	4.15	4.2	3.7
35–44	11.2	11.1	12.1	12.5	10.7	11.4
45–54	25.9	25.3	25.7	27.4	25.1	25.8
55–64	34.3	32.8	34.2	33	34.3	33.4
65+	24.9	27.3	24.5	23.0	25.9	25.5
**US region (%)**						
Northeast	62.6	65.2	62.3	57.6	56.2	62.7
Midwest	13.2	11.2	12.6	13.7	14.8	12.6
South	16.3	15.6	16.6	19.6	19.7	16.6
West	7.6	6.9	7.8	8.6	9.3	7.6
Other	0.4	1.1	0.7	0.5	0.1	0.6
**Health insurance type (%)**						
PPO	42.6	40.1	44.7	44.9	45.2	42.4
HMO	29.8	28.7	28.4	29.3	25.9	29.0
IND	10.1	8.2	8.7	8.9	12.9	9.3
POS	8.1	8.4	7.7	8.0	9.3	8.2
Medicare	7.2	12.3	8.2	5.8	3.7	8.7
Medicaid	0.7	1.1	1.0	1.3	1.0	0.9
Other	0.1	0.1	0.1	0.1	0.04	0.1
**Copayment at time of index Rx (%)**						
$0	8.2	7.9	7.3	6.7	8.5	7.8
$1–5	15.6	25.5	4.6	8.7	4.2	16.2
$6–10	43.1	33.1	16.4	26.8	17.5	33.5
$11–15	13.7	12.3	13.3	15.7	11.9	13.3
$16–20	12.5	12.0	23.3	21.6	23.4	15.1
$21–35	5.7	7.1	16.4	12.6	18.1	8.9
$35+	1.4	2.2	18.8	7.9	16.4	5.3
Total HC expenditures in preindex year	$1,771.15	$2,359.80	$1,864.22	$1,325.18	$1,572.84	$1,934.00
Any HC utilisation in preindex period	59.6	55.7	38.8	57.4	11.5	53.2

HC, healthcare; HMO, health maintenance organisation; IND, independent; MET, metformin; OAD, oral antidiabetes drug; OTH, other; POS, point of service; PPO, preferred provider organisation; SD, standard deviation; SFU, sulphonylurea; TZD, thiazolidinedione.

### HbA_1c_ testing and OAD treatment

HbA_1c_ tests were administered at least once to approximately 51.5% of patients, and fasting plasma glucose (FPG) testing was administered to 43% of patients (Table 2). Although most patients began OAD therapy at approximately the same time as their type 2 diabetes diagnosis, approximately 18% of patients initiated OAD treatment more than 1 year following diagnosis.

**Table 2:** HbA_1c_ testing and OAD treatment

	Index OAD Regimen					
	MET(*n* = 22,203) (%)	SFU(*n* = 18,441) (%)	TZD(*n* = 7663) (%)	SFU + MET (*n* = 5467) (%)	TZD + MET (*n* = 2356) (%)	Total(*n* = 56,130) (%)
Index regimen HbA_1c_ testing (Y/N, based on claims data)	54.6	48.3	49.7	51.8	53.9	51.5
Index regimen FPG testing (Y/N, based on claims data)	44.6	43.6	40.9	39	39	43
**Time (days) from diagnosis until first OAD therapy (%)**						
0	40.8	49.4	42.4	55.9	43.2	45.4
1–91	19.8	22.1	24	25.7	31.2	22
92–182	5.8	6.1	7.4	3.8	6.1	5.9
183–273	5	4.8	5	3	2.9	4.6
274–364	4.9	3.6	3.9	2.5	3.1	4.2
365+	23.7	14.2	17.3	9.1	13.6	17.8

FPG, fasting plasma glucose; HbA_1c,_ haemoglobin A1c; MET, metformin; OAD, oral antidiabetes drug; SFU, sulphonylurea; TZD, thiazolidinedione.

### Index OAD regimen duration, dosingand treatment gaps

For each index OAD regimen, patients’ treatment duration, dosing patterns and treatment gaps were evaluated.

The average index treatment duration was approximately 1 year for all treatment groups except the SFU + MET and TZD + MET groups, for which the average treatment length was 295 and 220 days respectively (Table 3). For all monotherapy index regimens, index regimens were titrated for 32.5% of patients at some point during therapy; 14.2% of patients receiving TZD therapy experienced a regimen titration. Patients taking SFU monotherapy were most likely to experience any up-titration (33.3%) or down-titration (21.2%), while patients receiving TZD or TZD + MET were the least likely to experience any up-titration (10.8% and 14.9% respectively) or down-titration (6.1% and 6.5% respectively). The majority (> 67%) of patients in each index group experienced no index regimen treatment gaps lasting ≥ 30 days.

**Table 3:** Index OAD regimen duration and dosing

	Index OAD Regimen					
Parameter	MET(*n* = 22,203)	SFU(*n* = 18,441)	TZD(*n* = 7663)	SFU + MET(*n* = 5467)	TZD + MET(*n* = 2356)	Total(*n* = 56,130)
Average index regimen duration (days)	355.3	373.9	333.8	295.3	220.3	346.9
% started on MED	2.3	3.2	7.4	45.5	35.1	7.4
Any regimen titration	34.50%	41.50%	14.20%	26.30%	17.30%	32.50%
**Up-titrations**						
% with 1+	30.7	33.3	10.8	22.3	14.9	27.3
Average time to first (days)	41	54.6	64	41.3	49.6	49
**Down-titrations**						
% with 1+	11.4	21.2	6.1	11.2	6.5	13.7
Average time to first (days)	54.6	65.5	85	52.7	55.1	62.2
Treatment gap(s) > 3 days (% with)	67.4	65.6	62	51.5	46.6	63.6
Treatment gap(s) ≥ 30 days (% with)	32.2	32.4	27.3	26.3	20.8	30.5

MED, maximum effective dose; MET, metformin; OAD, oral antidiabetes drug; SFU, sulphonylurea; TZD, thiazolidinedione.

### HbA_1c_ test utilisation and glycaemic control

Using claims data, an analysis of HbA_1c_ test utilisation was performed for each index group, including an evaluation of the number of tests performed, the timing of tests relative to the index regimen period and HbA_1c_ levels both at baseline and during the index regimen (Table 4).

**Table 4:** HbA_1c_ test utilisation and outcomes

	Index OAD regimen					
Parameter	MET(*n* = 22,203)	SFU(*n* = 18,441)	TZD(*n* = 7663)	SFU + MET(*n* = 5467)	TZD + MET(*n* = 2356)	Total(*n* = 56,130)
**Index regimen HbA_1c_ testing (based on claims data) (%)**						
Any test from 90 days priorto end of regimen	45.5	38.1	36.6	43.5	37.1	40.1
Within 90 days prestart	6.4	3.5	10.5	2.5	4.1	4.7
Within 90 days after start	24.1	19.5	18.9	22.6	24.1	21.7
Mean no. of tests	2.8	2.5	2.6	2.5	2.4	2.6
	(*n* = 1960)	(*n* = 1594)	(*n* = 849)	(*n* = 393)	(*n* = 252)	(*n* = 5048)
						
**Overall HbA_1c_results (subsample with laboratory values) (%)**						
Mean baseline HbA_1c_	7.7	8.6	8	8.9	8.3	8.2
Mean HbA_1c_ at 1st test ≥ 90 days after start	7	7.3	6.9	7.4	6.9	7.2
Baseline (days 0–89) %not controlled	59.8	78.1	65.4	79.6	67.5	68.5
During regimen (day ≥ 90) %not controlled	41.8	49.3	41.8	52.4	36.2	46.9

HbA_1c_, haemoglobin A1c; MET, metformin; OAD, oral antidiabetes drug; SFU, sulphonylurea; TZD, thiazolidinedione.

The number and timing of HbA_1c_ tests performed throughout the index OAD regimen period were similar across index groups. Approximately, 37% of TZD patients were administered an HbA_1c_ test at any time from 90 days prior to OAD initiation through the end of the regimen, compared with 45% of MET patients and 38% of SFU patients. HbA_1c_ tests were administered during the 90 days preceding the start of the index regimen for 4.7% of all patients, and 21.7% were tested during the 90 days following the start of a regimen.

*In the small subset of patients for whom laboratory data were available*, the percentage of patients demonstrating suboptimal or poor glucose control on HbA_1c_ testing decreased from 68.5% at baseline (days 0–89) across all index groups to 46.9% in patients who received HbA_1c_ testing ≥ 90 days after the start of the index regimen. At baseline, the average HbA_1c_ level for all groups was suboptimal (ranging from 7.7% among MET patients to 8.9% among SFU + MET patients). Mean HbA_1c_ at first test ≥ 90 days after the start of the index regimen showed the greatest reduction from mean baseline HbA_1c_ in the SFU + MET (−1.5) and TZD + MET (−1.4) groups, whereas the smallest reduction was observed among MET patients (−0.7); reductions in the SFU and TZD patients were −1.3 and −1.1 respectively. The TZD + MET cohort had the largest shift in patients moving from uncontrolled to controlled between the baseline HbA_1c_ test and the first test ≥ 90 days after initiating treatment.

### Regression analysis

The results of the logistic regression, indicating variables predictive of patients receiving any HbA_1c_ test are presented in Appendix S2. Several key predictors of reduced likelihood of HbA_1c_ testing were identified including SFU, TZD or TZD + MET treatment (vs. MET monotherapy), older age (65+ years), having Medicare as insurance and having a moderate insurance copayment ($11–20). Regional variances in HbA_1c_ testing likelihood were also identified, with the West region having the highest likelihood of testing. Amount of copayment was associated with likelihood of receiving an HbA_1c_ test, with patients with no copayment ($0) more likely to receive a test [odds ratio (OR), 2.45; 95% confidence interval (CI), 2.21–2.72] and those with higher copayments less likely, compared with patients with a $6–10 copayment. Any healthcare utilisation in the preindex period also increased the likelihood of testing (OR, 1.20; 95% CI, 1.15–1.24).

The results of the logistic regression, indicating variables associated with patients receiving an up-titration during index regimen are presented in Appendix S3. Factors associated with reduced likelihood of up-titration included TZD, SFU + MET, or TZD + MET treatment (compared with MET), younger age (18–34 years), Medicare as insurance and any preindex healthcare utilisation. The amount of copayment was also associated with likelihood of up-titration, with patients with copayment of $6–10 more likely to receive an up-titration than patients in any other copayment categories. Up-titration of initial OAD regimen was more likely for patients on SFU (vs. MET).

## Discussion

This study validated prior research that indicated that both inadequate HbA_1c_ testing and control, as well as a lack of timely OAD transitions and/or titrations are common in the US and appear to contribute substantially to inadequate blood glucose levels in patients with type 2 diabetes (7,13,14). These data also indicated substantial deviations in HbA_1c_ testing frequency compared with ADA and American College of Endocrinology/American Academy of Clinical Endocrinologists (ACE/AACE) clinical recommendations in place during the majority of the study period. In 2002, ADA and AACE implemented their first specific HbA_1c_ testing recommendations, calling for a minimum of quarterly and biannual testing respectively (22,23).

This study provides new insight regarding the timing of testing in clinical practice. Perhaps most surprising is an apparent deficit in FPG or HbA_1c_ assessment. For example, among patients with any HbA_1c_ testing, only 0.3% to 1.2% was tested in the 90 days before the start of their index regimen. This may indicate that pharmacological therapy was initiated without glucose control assessment and/or that other measurements (such as FPG) were used. Patients were most likely to receive HbA_1c_ testing within 90 days of starting their OAD regimen (22%); this is consistent, although not actually adherent, with then-current ADA recommendations that HbA_1c_ testing be repeated within 2–3 months of initiation to assess treatment efficacy (23).

Despite observable improvement in HbA_1c_ control among patients receiving any index OAD regimen, almost half failed to attain target glucose (HbA_1c_< 7.0%). Prior to OAD initiation, only approximately 32% of patients were at target; this proportion increased to 53.1% among patients who were tested at any time during OAD treatment. It should be noted that because laboratory values were only available for a modest subsample of the initial cohort, these results may not represent patterns of care for the entire cohort and should be interpreted with caution. However, these results correspond with a 2004 report evaluating US National Health and Nutrition Examination Survey (NHANES) outcomes (1988–1994 and 1999–2000), which found that only 37% of individuals with type 2 diabetes had HbA_1c_ levels < 7% (17). Likewise, two recent, large claims and managed care database studies evaluating new medication use in patients with type 2 diabetes corroborate that baseline population HbA_1c_ rates are typically elevated prior to treatment and do not improve subsequently to the extent that the majority of patients reach goal (6,24).

The multiple logistic regression model applied to analyse the discrete variable (yes/no, any HbA_1c_ testing) indicated only a moderate estimation of the observed variance; however, some novel information was identified regarding the characteristics of patients who do and do not receive HbA_1c_ testing. SFU drug use, older age (65+ years) and the use of inpatient hospital services and other services during the preindex period were predictive of less testing. Patients from the mid-Atlantic region (comprising the US states of NY, NJ and PA) represented a substantive proportion of the study sample (26.8%) and exhibited a > 50% decreased likelihood of receiving HbA_1c_ testing compared with the reference group (New England region, US), as well as with other US regions or the US as a whole. Variables associated with receiving HbA_1c_ testing include $0 copayment and having laboratory services performed during the preindex period, the latter of which is the single strongest predictor of testing.

The results of the logistic regression of likelihood of an up-titration during index regimen indicated that index regimens of TZD, SFU + MET, or TZD + MET treatment were less likely to be up-titrated, and those on SFU were more likely to be up-titrated. In addition, younger patients and those with Medicare were less likely to be titrated. Interestingly, the amount of copayment was also associated with likelihood of up-titration, but no clear trend was visible, as patients with copayments less than and more than $6–10 were less likely to receive an up-titration.

In addition to assess HbA_1c_ levels and testing frequency, this study was designed to evaluate OAD transition and titration patterns in the context of glucose control. Since the mid-1990s, the steady introduction of OADs with distinct mechanisms of action has made it increasingly feasible to maintain long-term glycaemic control prior to insulin initiation. The progressive nature of type 2 diabetes pathophysiology, however, currently requires regular, stepped treatment (1,5,6), and recognition of such treatment has led to the development of a series of algorithms intended to facilitate timely therapeutic progression (5,11,12). In particular, one 2007 guideline calls for stepping-up treatment within 2–3 months in patients not at goal (12). This new treatment approach may herald a new level of physician/patient vigilance, and places additional pressure on researchers and other stakeholders to more concretely define and address the barriers to effective glycaemic control.

A growing body of research has indicated that successful OAD management is predicated on physician adherence to treatment algorithms and attention to patient follow-up (6,7,13). A series of studies has identified a link between clinical inertia (in which health providers delay or fail to start step-up therapy as recommended) and inadequate glucose control in well-managed healthcare organisations (6,7). Other research identified poor patient adherence as a key contributor to poor glucose control (9,10). Most recently, a study by Parchman et al. (8) identified the competing demands placed on primary care physicians during office visits as the strongest predictor of timely medication adjustments in type 2 diabetes; based on this, the authors posited that clinical inertia provides an incomplete explanation for the complexity of the problem (8).

There were several limitations to this analysis. There are a myriad of potential reasons for OAD discontinuation (such as adverse events), which could not be captured using these claims data. Self-monitoring of blood glucose or in-office glucose tests was not considered as part of this analysis, but may have been alternatives to HbA_1c_ testing in the assessment of patient glucose control. This analysis does not include the more recently introduced drug regimens [i.e. glucagon-like peptide-1s (GLP-1s), dipeptidyl peptidase-4 (DPP-4) inhibitors]. Information on patient ethnicity was not available in the IHCIS data set, and there was limited availability of laboratory values data for a subsample of the initial cohort. These and other relevant factors that are not captured in this data set may have had a significant impact on the outcomes studied here; therefore our findings should not be considered as proof of any specific hypothesis, but rather as confirmation of associations between certain treatment patterns and outcomes.

This study confirmed that glucose control in the US was inadequate between 2001 and 2006, and indicated that this may in part be because of patients not being transitioned to new OADs in a stepwise fashion and/or not receiving appropriate titration of current OAD regimens. A possible explanation may be unwillingness by physicians to either titrate or add new OADs caused by a perceived lack of efficacy or tolerability. The current findings indicated that only approximately 20–34% of patients received any index OAD titrations. Current type 2 diabetes management guidelines focus on adding new OADs to patient regimens and only briefly address the potential therapeutic value of up-titrating existing OADs, in particular MET, to maximum effective dose (MED) (11). This issue has remained overlooked in the literature even though MEDs have been identified for most OADs (25), and inadequate MED titration has been documented as a potential contributor to inadequate glucose control (13,14). Further research is needed into the clinical decision process for whether (and how) to intensify OAD treatment regimens, as well as the outcomes associated with each option.

## Suppporting information

**Appendix S1** Oral antidiabetes classes and medications (26).

**Appendix S2** Logistic regression results: variables associated with patients receiving any HbA_1c_ testing.

**Appendix S3** Logistic regression results: variables associated with patients receiving any OAD up-titration.
