# Supplementary material for: Drug titration patterns and HbA1c levels in type 2 diabetes
Source: Int J Clin Pract. 2009 Jul;63(7):1008–16. doi: 10.1111/j.1742-1241.2009.02094.x (PMC2984545; doi:10.1111/j.1742-1241.2009.02094.x)
Supplement: Supplementary file 1 [file ijcp0063-1008-SD1.doc]

**Appendix A:** Oral antidiabetes classes and medications [26]

(For Web only)

| **Class** | **Generic Name / Second Agent in Combination** | **Brand Name*** | **Doses (mg)** | **Maximum dose (mg)** | **Maximum effective  dose (mg)** | **HbA**_1c_ **reduction (%)**^a^ |
| --- | --- | --- | --- | --- | --- | --- |
|  |  |  |  |  |  |  |
| Sulphonylurea | glyburide^c^ | Diabeta® | 1.25, 2.5, 5 | 10 bid | 10 qd | 1.5-2.0 |
|  | glipizide^c^ | Glucatrol® | 5, 10 | 20 bid | 10 qd-bid | 1.5-2.0 |
|  | glipizide GITS^†^ | Glucatrol-XL® | 2.5, 5, 10 | 20 qd | 5-20 qd | 1.5-2.0 |
|  | glimepiride | Amaryl® | 1, 2, 4 | 8 qd | 4 qd | 1.5-2.0 |
| Non-sulphonylurea insulin secretagogue | repaglinide | Prandin® | 0.5, 1, 2 | 4 tid | 2 tid | 1.5-2.0 |
|  | nateglinide | Starlix® | 60, 120 | 120 tid | 120 tid | 0.5-1.0 |
| Biguanide | metformin^c^ | Glucophage® | 500, 850, 1000 | 850 tid | 1000 bid | 1.5-2.0 |
|  | metformin-XR | Glucophage®-XR | 500 | 2000 qd | 2000 qd | 1.5-2.0 |
| Thiazolidinedione | rosiglitazone | Avandia® | 4, 8 | 8 qd, 4 bid | 4 bid | 1.5 |
|  | pioglitazone | Actos® | 15, 30, 45 | 45 qd | 45 qd | 1.5 |
| Alpha-glucosidase inhibitor | acarbose | Precose® | 50, 100 | 100 tid | 50 tid | 0.5-1.0 |
|  | miglitol | Glyset® | 25, 50, 100 | 100 tid | 100 tid | 0.75-1.2 |
| Single-pill combination with metformin | glyburide | Glucovance® | 1.25/250, 2.5/500, 5/500 | 5/500, 2 bid | 2.5/500, 2 bid^b^ | 1.3^c^ |
|  | glipizide | Metaglip™ | 2.5/250, 2.5/500, 5/500 | 5/500, 2 bid | 5/500, 2 bid^b^ | 2.1^d^ |
|  | rosiglitazone | Avandamet® | 1/500, 2/500, 4/500 | 2/500, 2 bid | 2/500, 2 bid^b^ | N/A^e^ |

^a^Approximate haemoglobin A1c (HbA_1c_) reduction vs placebo at maximally effective dose, except where indicated.

^b^Represents equivalent maximally effective doses of components.

^c^Mean dose 4.1 mg/824 mg daily (Glucovance® package insert).

^d^Mean dose 7.4 mg/1477 mg daily (Metaglip™ package insert).

^e^No clinical efficacy trials conducted.

*Actos® (Takeda); Amaryl®, Diabeta® (Sanofi-Aventis); Avandia®, Avandamet® (GlaxoSmithKline); Glucatrol®, Glucatrol-XL®, Glyset® (Pfizer); Glucophage®, Glucophage®-XR, Glucovance®, Metaglip™ (Bristol-Myers Squibb); Prandin® (Novo Nordisk); Precose® (Bayer); Starlix® (Novartis).

^†^GITS, gastrointestinal therapeutic system.

*Source*: Adapted from Sheehan MT. Current therapeutic options in type 2 diabetes mellitus: A practical approach. *Clin Med Res* 2003; **1**: 189–200 (Table 1)

**Appendix B** Logistic regression results: variables associated with patients receiving any HbA_1c_ testing

(For Web only)

| *Parameter (Referent)* | *Odds*  *ratio* | *95% CI* | *p-value* |
| --- | --- | --- | --- |
| Intercept | -- | -- | <0.0001 |
| Treatment group (vs MET)  SFU  TZD  SFU + MET  TZD + MET | 0.767  0.857  0.906  0.918 | 0.736-0.798  0.810-0.906  0.852-0.963  0.840-1.004 | <0.0001  <0.0001  0.0015  0.0624 |
| Age (vs 55–64 years)  18–34  35–44  45–54  65+ | 0.973  0.945  0.999  0.814 | 0.887-1.068  0.891-1.001  0.955-1.044  0.814-0.860 | 0.5649  0.0540  0.9541  <0.0001 |
| US region (vs West)  Midwest  Northeast  South  Other | 0.665  0.430  0.701  0.513 | 0.609-0.726  0.399-0.463  0.645-0.763  0.411-0.640 | <0.0001  <0.0001  <0.0001  <0.0001 |
| Gender (vs male)  Female | 1.044 | 1.009-1.080 | 0.0139 |
| Insurance (vs PPO)  HMO  IND  Medicare  Medicaid/Other  POS | 1.156  1.167  0.860  0.879  1.417 | 1.108-1.206  1.091-1.248  0.796-0.930  0.771-1.002  1.327-1.512 | <0.0001  <0.0001  0.0001  0.0532  <0.0001 |
| Cost (vs $6–$10)  $0–$5  $11–$15  $16–$20  $21–$35  $36+ | 1.356 0.681  0.873 0.953  0.936 | 1.295-1.420  0.644 -0.720  0.828-0.920  0.892-1.017  0.861-1.017 | <0.0001  <0.0001  <0.0001  0.1445  0.1179 |
| Insulin (vs no) | 0.914 | 0.837-0.999 | 0.0472 |
| HC utilisation (vs none) | 1.196 | 1.153-1.241 | <0.0001 |

CI, confidence interval; HbA_1c_, haemoglobin A1c; HC, healthcare; HMO, health maintenance organisation; IND, independent; MET, metformin; POS, point of service; PPO, preferred provider organisation; SD, standard deviation; SFU, sulphonylurea; TZD, thiazolidinedione.

**Appendix C** Logistic regression results: variables associated with patients receiving any OAD
up-titration

(For Web only)

| *Parameter (Referent)* | *Odds ratio* | *95% CI* | *p-value* |
| --- | --- | --- | --- |
| Intercept |  |  | <0.0001 |
| Treatment group (vs MET)  SFU  TZD  SFU + MET  TZD + MET | 1.147  0.296  0.010  0.010 | 1.099-1.197  0.273-0.321  0.006-0.014  0.005-0.018 | <0.0001  <0.0001  <0.0001  <0.0001 |
| Age (vs 55–64 years)  18–34  35–44  45–54  65+ | 0.871  1.069  1.034  0.973 | 0.777-0.976  0.998-1.144  0.981-1.090  0.912-1.038 | 0.0175  0.0582  0.2130  0.4051 |
| US region (vs West**)**  Midwest  Northeast  South  Other | 1.009  1.171  0.987  0.962 | 0.909-1.119  1.073­-1.278  0.893-1.091  0.740-1.250 | 0.8713  0.0004  0.7956  0.7700 |
| Gender (vs male)  Female | 1.028 | 0.987-1.070 | 0.1806 |
| Insurance (vs PPO)  HMO  IND  Medicare  Medicaid/Other  POS | 0.982  1.045  0.899  0.855  0.932 | 0.934-1.032  0.964­-1.133  0.820-0.984  0.726-1.007  0.863-1.008 | 0.4694  0.2815  0.0210  0.0606  0.0769 |
| Cost (vs $6–$10)  $0–$5  $11–$15  $16–$20  $21–$35  $36+ | 0.885  0.795  0.913  0.724  0.764 | 0.841-0.931  0.745-0.849  0.856-0.974  0.664-0.789  0.672-0.868 | <0.0001  <0.0001  0.0058  <0.0001  <0.0001 |
| Insulin (vs no) | 0.949 | 0.852-1.057 | 0.3405 |
| HC utilisation (vs none) | 0.946 | 0.906-0.989 | 0.0141 |

CI, confidence interval; HC, healthcare; HMO, health maintenance organisation; IND, independent; MET, metformin; OAD, oral antidiabetes drug; POS, point of service; PPO, preferred provider organisation; SFU, sulphonylurea; TZD, thiazolidinedione
